# Evolutionary Diversification of New Caledonian *Araucaria*

**DOI:** 10.1371/journal.pone.0110308

**Published:** 2014-10-23

**Authors:** Mai Lan Kranitz, Edward Biffin, Alexandra Clark, Michelle L. Hollingsworth, Markus Ruhsam, Martin F. Gardner, Philip Thomas, Robert R. Mill, Richard A. Ennos, Myriam Gaudeul, Andrew J. Lowe, Peter M. Hollingsworth

**Affiliations:** 1 Royal Botanic Garden Edinburgh, Edinburgh, United Kingdom; 2 University of Edinburgh, Institute of Evolutionary Biology, Edinburgh, United Kingdom; 3 UMR CNRS MNHN UPMC EPHE 7205 Institut de Systématique, Evolution, Biodiversité, Muséum National d'Histoire Naturelle, Paris, France; 4 Australian Centre for Evolutionary Biology and Biodiversity, Environment Institute, School of Earth and Environmental Science, University of Adelaide, Adelaide, Australia; University of Arkansas, United States of America

## Abstract

New Caledonia is a global biodiversity hotspot. Hypotheses for its biotic richness suggest either that the island is a ‘museum’ for an old Gondwana biota or alternatively it has developed following relatively recent long distance dispersal and *in situ* radiation. The conifer genus *Araucaria* (Araucariaceae) comprises 19 species globally with 13 endemic to this island. With a typically Gondwanan distribution, *Araucaria* is particularly well suited to testing alternative biogeographic hypotheses concerning the origins of New Caledonian biota. We derived phylogenetic estimates using 11 plastid and rDNA ITS2 sequence data for a complete sampling of *Araucaria* (including multiple accessions of each of the 13 New Caledonian *Araucaria* species). In addition, we developed a dataset comprising 4 plastid regions for a wider taxon sample to facilitate fossil based molecular dating. Following statistical analyses to identify a credible and internally consistent set of fossil constraints, divergence times estimated using a Bayesian relaxed clock approach were contrasted with geological scenarios to explore the biogeographic history of *Araucaria*. The phylogenetic data resolve relationships within Araucariaceae and among the main lineages in *Araucaria*, but provide limited resolution within the monophyletic New Caledonian species group. Divergence time estimates suggest a Late Cretaceous-Cenozoic radiation of extant *Araucaria* and a Neogene radiation of the New Caledonian lineage. A molecular timescale for the evolution of Araucariaceae supports a relatively recent radiation, and suggests that earlier (pre-Cenozoic) fossil types assigned to *Araucaria* may have affinities elsewhere in Araucariaceae. While additional data will be required to adequately resolve relationships among the New Caledonian species, their recent origin is consistent with overwater dispersal following Eocene emersion of New Caledonia but is too old to support a single dispersal from Australia to Norfolk Island for the radiation of the Pacific *Araucaria* sect. *Eutacta* clade.

## Introduction

New Caledonia, in the tropical south-western Pacific, is noted for a remarkably rich biota with outstanding levels of endemism [1]. The island has been considered both a ‘museum’ for phylogenetic relicts and a natural laboratory for the study of island radiations [2]. A long held view is that New Caledonia (as part of Zealandia) was rifted from the eastern Australian margin some 80 million years before present (Ma), carrying a complement of Gondwanan lineages that have since evolved there in relative isolation [3]. Alternatively, geological reconstructions for the region indicate lengthy deep-water marine transgressions (Palaeocene-Eocene) with limited evidence of a continual landmass prior to the emersion of New Caledonia in the Late Eocene-Oligocene (c. 37 Ma) [4]. This implies that dispersal events must be central in the assembly of the modern biota [2]. Phylogenetic and molecular dating methods, within the context of a well-constrained geological scenario, can be used to explore these alternative biogeographic scenarios [5]. With respect to New Caledonia such studies provide strong support for the latter view [2], [6].

The conifer genus *Araucaria* Juss. (Araucariaceae) provides a good model to explore alternative hypotheses regarding the age, origins and diversification of the New Caledonian biota [7], [8]. *Araucaria* has been considered an old lineage [9] and for instance, fossil data from both the Northern and Southern Hemisphere of the early Mesozoic (Jurassic, c. 190 Ma, or earlier) have been taken to indicate that at least some of the extant sections of *Araucaria* had begun to diversify and were widespread by this time. Thirteen of the 19 recognised *Araucaria* species are now endemic to New Caledonia, with the other species occurring in eastern Australia and New Guinea (3 spp., 1 shared), Norfolk Island (1 sp.) and southern South America (2 spp.). In light of these data extant *Araucaria* have been considered a relict of a formerly widespread Gondwanan distribution [10].

A key question is the biogeographic origin of the New Caledonian *Araucaria* – an old lineage isolated by vicariance that has diversified *in situ* or a relatively recent radiation following long distance dispersal? There have been some indirect tests of these hypotheses. Setoguchi et al. [8] used plastid *rbcL* sequence data to infer the phylogeny of Araucariaceae and found support for the monophyly of the New Caledonian *Araucaria*. In this study, however, the morphological species were characterised by very low levels of DNA sequence divergence suggestive of a recent radiation. Gaudeul et al. [7] also argued for a recent diversification of the New Caledonian *Araucaria* based on a low ratio of among versus within species genetic divergence using AFLP markers. In the context of these findings it has been proposed that the origin of this lineage may be maximally constrained by the age of Norfolk Island (c. 1000 km to the south of New Caledonia) [2], a volcanic island that in its present form emerged c. 3.7 Ma. This island is home to the sister species (*A. heterophylla* (Salisb.) Franco; Norfolk Island Pine) of the New Caledonian *Araucaria* lineage [8], [11]. However, the use of island age to date island-endemic lineages can be problematic as these taxa could represent historically widespread lineages that have been isolated by extinction [12]. Limitations of the molecular clock have also been well-characterised [13] and there may be a poor correlation between levels of DNA sequence divergence and divergence times if rates have been slow relative to related taxa. Furthermore, finding low levels of divergence among molecular markers does not rule out the possibility of an ancient lineage and a recent diversification in the absence of a timescale for the origin of the New Caledonian lineage [14]. Recent analyses of araucarian and conifer divergence times [15]–[19] for the most part suggest a relatively recent radiation of extant Araucariaceae and *Araucaria* but as yet there has been no formal attempt to date the diversification of *Araucaria* on New Caledonia.

In the present study, we develop a phylogenetic estimate for *Araucaria* using rDNA internal transcribed spacer 2 (ITS2) sequences and a mix of 11 coding and non-coding plastid data. We include a complete sampling of species and several accessions of each of the 13 New Caledonian representatives. Our overall aims are: (1), to clarify relationships among *Araucaria*, particularly the New Caledonian species; (2), using well-constrained fossil dates and molecular clock methods, to estimate a timescale for *Araucaria* evolution focussing on the origins of the New Caledonian radiation.

## Methods

### Plant material and DNA extractions

Collection, voucher and permit details of all samples used for the phylogenetic analyses are listed in Table S1. Permits allowed for the collection of samples from threatened species and for collection of samples from within protected areas. CITES permits were obtained for *Araucaria araucana*. DNA was extracted using the Qiagen DNeasy Mini Plant Kit following the manufacturer's protocol.

### PCR amplification and sequencing

DNA sequences were obtained from 11 chloroplast regions (*matK*, *rcbL*, *rpoB*, *rpoC1*, *atpF-atpH*, *atpH-atpI*, *rps12-rpl20*, *trnC-ycf6*, *trnH-psbA*, *trnS-trnfm*, *trnS-trnG*) and one nuclear region (ITS2). For a subset of 21 taxa (nine *Agathis* species, and outgroup taxa including a single representative each of the genera *Pinus* L. (Pinaceae), *Taxus* L. (Taxaceae), *Cryptomeria* D.Don (Cupressaceae), *Taxodium* Rich. (Taxodiaceae), *Lepidothamnus* Phil., *Halocarpus* Quinn, *Falcatifolium* de Laub., *Dacrycarpus* de Laub., *Phyllocladus* Rich. ex Mirb., *Podocarpus* L'Hér. ex Pers., *Retrophyllum* C.N.Page and *Sundacarpus* (J.Buchholz and N.E. Gray) C.N. Page (all Podocarpaceae)) we assembled a data set comprising *rbcL, matK* and non-coding plastid regions *atpF-atpH* and *rps12-rps20* to supplement the *Araucaria* data for molecular dating analyses (see below). These regions were chosen because they could be unambiguously aligned in all taxa.

PCR primers and conditions for *matK*, *rcbL*, *rpoB*, *rpoC1*, *atpF-atpH* and *trnH-psbA* are detailed in Appendix 4 of the supplementary material of [20]. For the remaining regions primer sequences were taken from the following publications: *atpH-atpI* [21], *rps12-rpl20* and *trnC-ycf6* [22], *trnS-trnfm* [23], *trnS-trnG* [24] and ITS2 [25].

PCR reactions for *atpH-atpI*, *rps12-rpl20*, *trnC-ycf6*, *trnS-trnG* and ITS2 were performed in volumes of 25 µl using the following protocol: 1xbuffer (Bioline, London, UK), 0.2 mM dNTPs, 2.5 mM MgCl_2_, 0.3 µM of each forward and reverse primer, (4% Dimethyl Sulfoxide, DMSO, ITS2 only), 0.04 U BioTaq (Bioline, London, UK) and 1 µl of unquantified DNA. The mixture was then cycled through the profile: 2 min at 95°C, 40 cycles of 30 sec at 95°C, 30 sec at 50°C and 1 min at 72°C, ending with 10 min at 72°C to complete extension and subsequent storage at 4°C.

PCR reactions for *trnS-trnfm* followed this protocol (25 µL): 1x buffer (Bioline, London, UK), 0.2 mM dNTPs, 2.5 mM MgCl_2_, 0.3 µM of each forward and reverse primer, 2 M Betaine, 0.03 U BioTaq (Bioline, London, UK) and 2 µl of unquantified DNA. The mixture was then cycled through the following profile: 4 min at 94°C, 30 cycles of 45 sec at 94°C, 45 sec at 62°C and 2 min at 72°C, ending with 10 min at 72°C and subsequent storage at 4°C.

PCR products were cleaned using 2 µl exonuclease I and shrimp alkaline phosphatase (ExoSAP) for 5 µl of product and sequenced in two reactions using each of the two PCR primers, following Big Dye v. 3.1 chemistry (Applied Biosystems, Warrington, UK). Sequences were then assembled and aligned using Sequencher v. 3.7 (GeneCodes Corp., Ann Arbor, Michigan, USA).

### Phylogeny

*Araucaria* phylogeny was inferred separately for the plastid (53 taxa), ITS2 (48 taxa) and a combined plastid and ITS2 DNA sequence data set (48 taxa) using both maximum likelihood (ML) and Bayesian inference (BI) optimisation criteria (Table S1; vouchers).

We used GARLI 0.951 [26] for the ML analyses with a general time reversible model of sequence evolution, gamma distributed rate variation and a proportion of invariant sites (GTR+I+Γ). Clade support was assessed using 250 non parametric bootstrap (BS) pseudoreplicates.

Bayesian inference was performed using MrBayes 3.1.2 [27], [28]. For the plastid and combined data sets we used a mixed model approach with GTR+I+Γ model parameters estimated separately for each locus in the concatenated matrices. For the analysis of the ITS2 sequences a GTR+I+Γ model was used. For each analysis we performed four independent runs of 2×10^6^ generations, sampling topology and parameter values every hundredth generation, with default (flat) priors, a random starting tree and four starting chains (one cold, three heated). Convergence between runs was assessed relative to the variance in parameter estimates (average standard deviation of split frequencies <0.01; Ronquist 2005) and by inspection of parameter estimates in Tracer v1.5 [29]. Tracer was used to estimate the burn in proportion, and 50% majority rule consensus topologies were generated from the pooled post-burn in topologies from the four independent runs.

### Molecular Dating

#### Molecular data

To estimate topology and divergence times for Araucariaceae, we assembled a data set comprising partial sequences of the plastid genes *rbcL, matK* and non-coding plastid regions *atpH-atpI* and *rps12-rps20* for 45 taxa). Taxon sampling includes all of the 19 currently recognised species of *Araucaria*, 11 species of *Agathis* Salisb., *Wollemia* (monotypic), and outgroup taxa including Cupressaceae, Taxaceae, Podocarpaceae and Pinaceae (Table S1).

#### Fossil constraints

**Figure 1:**
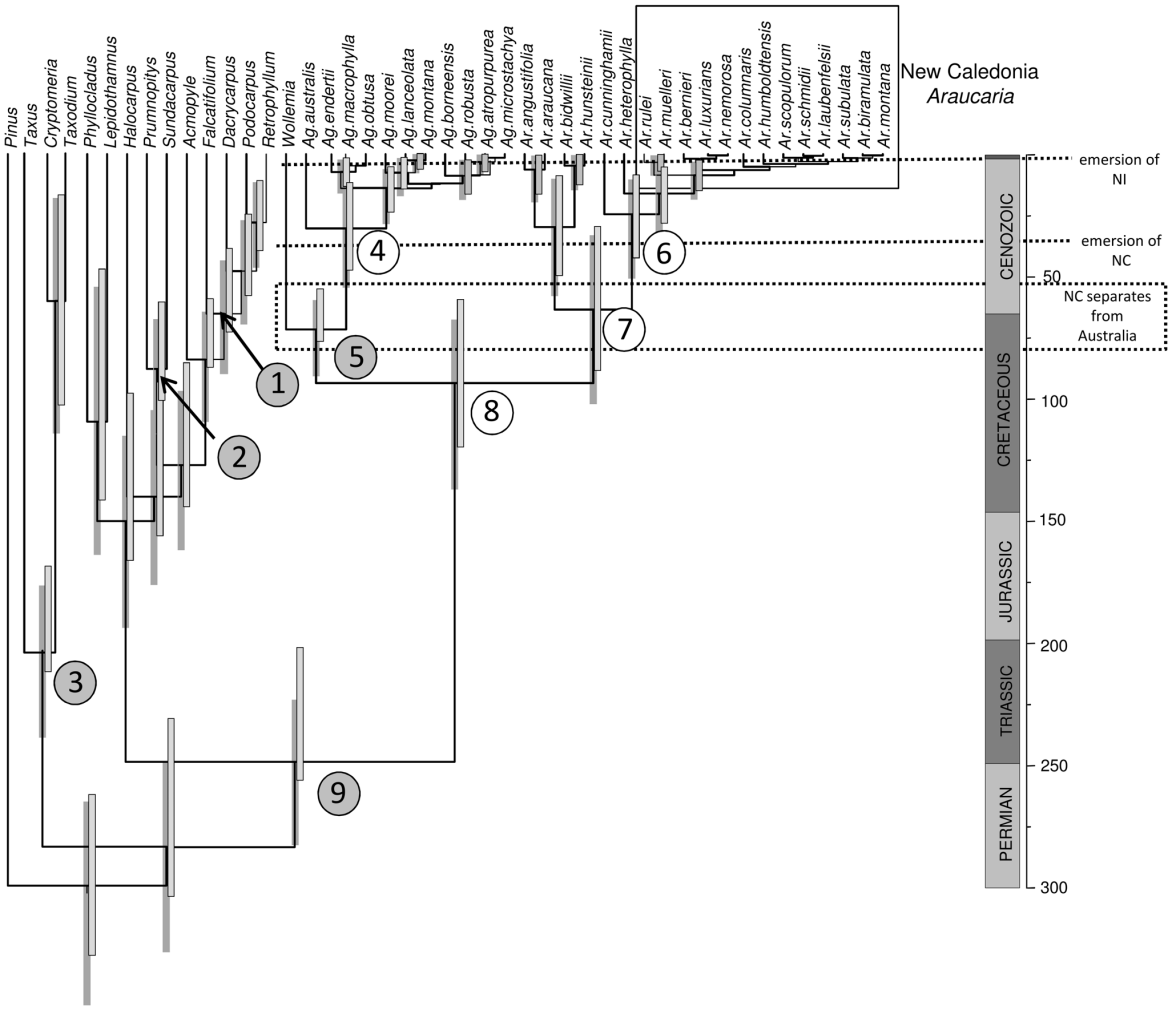
Divergence time estimates for Araucariaceae. The topology presented is the maximum credibility tree (median node heights) obtained by pooling four separate runs under two constraint scenarios (see Table 1, and text for details). Circles indicate nodes that were included in the assessment of candidate nodal constraints, and the dark shaded circles indicate nodes that were constrained for the BRC analyses (see Table 1 for details). Node bars (nodes with a posterior probability ≥0.75) indicate the 95% HPD of divergence times for the two prior scenarios: left, log normal prior mean  =  minimum fossil age +50%; right, log normal prior mean  =  minimum fossil age +10% (NC, New Caledonia; NI, Norfolk Island).

Five fossil dates were used to calibrate molecular evolutionary rates amongst Araucariaceae. These comprise two fossil constraints that have been assigned to the Araucariaceae crown group, two within the Podocarpaceae as well as a constraint upon the age of the Cupressaceae *sensu lato* (Table 1). The araucarian fossils include the oldest unequivocal macrofossil remains of *Agathis* from south-eastern Australia of the Late Palaeocene (*A. vittata* R.S.Hill et al., c. 55 Ma) [30] and a recently described bract-scale complex from the Lower Jurassic (c. 190 Ma) of eastern North America, which has been proposed as the earliest known occurrence of *Araucaria* section *Eutacta* (Link) Endl. [31]. It should be noted that this fossil age is not inconsistent with several other well documented fossil types (e.g. *Araucaria mirabilis* (Spegazzini) Windhausen, *A. sphaerocarpa* Carruthers) that have suggested affinities within extant *Araucaria* [32]. Several extant podocarp lineages have reliable macrofossil records including *Prumnopitys* Phil. [33], [34] and *Acmopyle* Pilg. [33], [35]. We used macrofossil remains from K-T boundary exposures (c. 65 Ma) of New Zealand [34] to constrain the crown group age for *Prumnopitys sensu lato* (most recent common ancestor, hereafter MRCA, of *Prumnopitys* + *Sundacarpus*; see [36]) and defined a minimum age of 55 Ma to constrain the *Acmopyle* stem (MRCA *Podocarpus + Retrophyllum + Dacrycarpus* + *Falcatifolium* + *Acmopyle*, based on *A. florinii* R.S.Hill and Carpenter). The fossil taxon *Austrohamia minuta* Escapa, Cúneo and Axsmith, from the Middle-Late Jurassic has been placed within Cupressaceae *sensu lato* following a cladistic analysis of morphological characters from fossil and living taxa [37]. On this basis we assumed a minimum age of 160 Ma to constrain the Cupressaceae stem (MRCA Cupressaceae s.l. + Taxaceae) (Table 1; Figure 1).

**Table 1:** Details of fossil constraints used to estimate araucarian divergence times (Prior 1, log normal prior mean  =  minimum fossil age +10%; Prior 2, log normal prior mean  =  minimum fossil age +50%) and empirical scaling factor (S*_i_*) estimates inferred from the unconstrained BRC analyses of the DNA sequences.

Node	Fossil	Minimum age constraint (Ma)	S*_i_* (mean[95% PD])	Prior 1	Prior 2
**1**	*Acmopyle florinii*	55	**186 (250–133)**	**0.5, 2**	**0.5, 3.3**
**2**	*Prumnopitys* ‘Mt. Somers’	60	**175 (247–170)**	**0.5, 2**	**0.5, 3.3**
**3**	*Austrohamia minuta*	160	**284 (413–210)**	**0.5, 2.8**	**0.5, 4.4**
4			784 (1186–415)	-	-
**5**	*Agathis vittata*	55	**368 (565–198)**	**0.5, 2**	**0.5, 3.3**
6			3083 (5426–1305)	-	-
7	‘araucarian bract scale complex’ (cf. *Araucaria* section *Eutacta*)		1260 (1928–698)	-	-
8		190	842 (1191–488)	-	-
**9**			**221 (264–196)**	**0.5, 3.2**	**0.5, 4.5**

Node numbers are as per Figure 1. Values highlighted in bold were selected on the basis of their S*_i_* values for the molecular dating analyses. PD, marginal posterior distribution of S*_i_*; SD, standard deviation of the log normal prior. Values in the Prior1 and Prior 2 column denote SD and log normal mean, respectively.

#### Assessment of fossil constraints

We adopted the approach of Dornburg et al. [38] to provide an assessment of the palaeontological data described above. These authors extend the methods of Marshall [39], which use minimum node age estimates derived from the fossil record and an uncalibrated ultrametric tree to bracket absolute divergence times from molecular phylogenies. Given that for a particular lineage the known fossil record is generally incomplete, Marshall [39] provides an approach to estimate the coverage of a fossil assigned to that clade (empirical scaling factor, S*_i_*, i.e. the proportion of the true duration of the lineage represented by its oldest known fossil) which can be used to estimate the true scaling factor, S, i.e. the actual time of divergence of the root. To incorporate uncertainty in S*_i_* estimates, Dornburg et al. [38] sample a distribution of ultrametric tree topologies and relative branch lengths. Rather than applying an age bracket to the single fossil with the highest coverage [39], they identify a set of internally consistent fossil constraints based upon the S*_i_* estimates and this set of consistent calibrations is incorporated in molecular dating analyses to derive absolute estimates of node heights [38].

We estimated the posterior distribution of uncalibrated ultrametric trees (i.e. branch lengths in units of substitutions/site) from the molecular data using the Bayesian relaxed clock (BRC) application BEAST (v.1.6.1) [40] with a GTR+I+Γ model of sequence evolution, a Yule prior on branch rates and an uncorrelated log normal relaxed clock model [41]. For these analyses two independent Markov-chain Monte Carlo runs were performed, each of 4×10^7^ steps (sampling topology and parameter values every 5000 steps). Tracer v1.5 [29] was used to assess convergence between runs and estimate an appropriate burn-in proportion, the mean and 95% highest posterior density (HPD) of parameters sampled from the posterior distribution of the combined runs, and to ensure that the effective sample size was sufficient to provide reasonable estimates of model parameter variance (i.e.>200).

For each fossil constraint (Table 1) S*_i_* was estimated using Equation 1 of Marshall [39] and the distribution of S*_i_* values was estimated from 1000 topologies sampled from posterior distribution of uncalibrated ultrametric trees. In order to assess the internal consistency of the fossil constraints, we simultaneously compared the interval encompassing 95% of the values for the fossil with the highest S*_i_* estimates (i.e. the highest coverage) against all other S*_i_* intervals for each fossil. The consistent fossil set is defined as those with 95% S*_i_* intervals that overlap the interval with the highest S*_i_* values [38].

Where possible, we assessed the relative placement of the fossil age within a lineage (i.e. crown or stem node). For three of the constraints, taxon sampling (*Acmopyle* and Cupressaceae) and/or phylogenetic uncertainty (*Prumnopitys* s.l.) excluded this possibility, but for the *Agathis* fossil age, we compared crown and stem node placement (MRCA *Agathis* + *Wollemia*) (nodes 4 and 5, respectively; Table 1, Figure 1). For the *Araucaria* section *Eutacta* fossil age we considered alternative placements on the section *Eutacta* crown (node 6), *Araucaria* crown (node 7), Araucariaceae crown (node 8), and on the Araucariaceae stem (MRCA of Araucariaceae + Podocarpaceae; node 9). This is in the light of questions, on a number of grounds [8], [11], [15], [42] of generic and/or sectional affinities of Early Mesozoic araucarians.

We placed a caveat on the selection of the fossil with the highest coverage. Fossil prepollen of *Potonieisporites* D.C.Bhardwaj, a monosaccate type that was produced by walchian conifers, is known from at least as early as the Langsettian (Upper Carboniferous >313–314 Ma) from Scotland [43] while macrofossils of the walchian conifer *Hermitia* Kerp and Clement-Westerhof are known from at least as early as the Asturian ( =  Westphalian D, 308–306 Ma) of the Netherlands [44]. Extant conifer families have fossil records extending from the Triassic [45], [46] providing an expectation on the ‘upper’ distribution of reasonable S*_i_* values. Estimates that consistently exceeded the age of the earliest known conifers were treated as outliers.

#### Divergence time estimates

We used the consistent set of fossils identified following the procedure outlined above to estimate araucarian divergence times with BEAST. For each fossil datum, we defined a log normal prior probability distribution on the age of the fossil constrained node, with a hard minimum age equivalent to the age of the associated fossil. The other parameters of the log normal distribution are the mean and standard deviation. For all analyses, the standard deviation was set to 0.5, and to model taphonomic bias of the fossil record, the log normal prior mean was set at c. 10% and 50% older than each minimum fossil age, in separate sets of analyses (Table 1). At the highest value of the mean, the upper bound of the 95% confidence interval (CI) of the log normal constraint priors included values estimated using Marshall's equation 11 [39] approach for estimating a 95% CI for the true time of origin of a lineage, given its oldest known fossil (with *n* = 5, i.e. the number of fossils included in the consistent set of fossil calibrations) [38]. For each value of the log normal prior mean, two separate BEAST analyses were performed, with model parameters and settings as outlined above. For each mean value, the two separate runs were combined after excluding the burn in fraction, and topologies and parameter values were summarised on the ‘maximum credibility’ tree using Tree Annotator v. 1.6. [40]. For these analyses we additionally constrained the root age with a log normal prior with a mean of c. 300 Ma and a 95% CI ranging from 260–350 Ma approximating the age of stem group conifers [46].

## Results

### Sequence data

The alignment of the 11 used plastid regions and the nuclear ribosomal ITS2 comprised 9970 bp and 500 bp, respectively, totalling 10470 bp in the combined dataset. For all *Araucaria* samples (except *A.schmidii*), *Agathis moorei* (Lindl.) Mast., *Agathis lanceolata* Warb., *Wollemia nobilis* W.G.Jones et al., *Prumnopitys ferruginoides* (Compton) de Laub. and *Acmopyle pancheri* (Brongn. and Gris) Pilg., sequencing was successful for all individuals and regions (Table S2). Certain regions could not be obtained for *Prumnopitys ferruginoides* (*rpoB*, *trnH-psbA, trnS-trnfm*), *Acmopyle pancheri* (*trnH-psbA*), *A. schmidii* de Laub. (ITS2), *Taxodium distichum* (*atpF-atpH*), *Podocarpus gnidioides* (*rps12-rpl20*) as well as *Lepidothamnus fonkii* and *Retrophyllum rospigliosii* (both *atpF-atpH* and *rps12-rpl20*) (Table S2).

### Phylogeny of *Araucaria*

**Figure 2:**
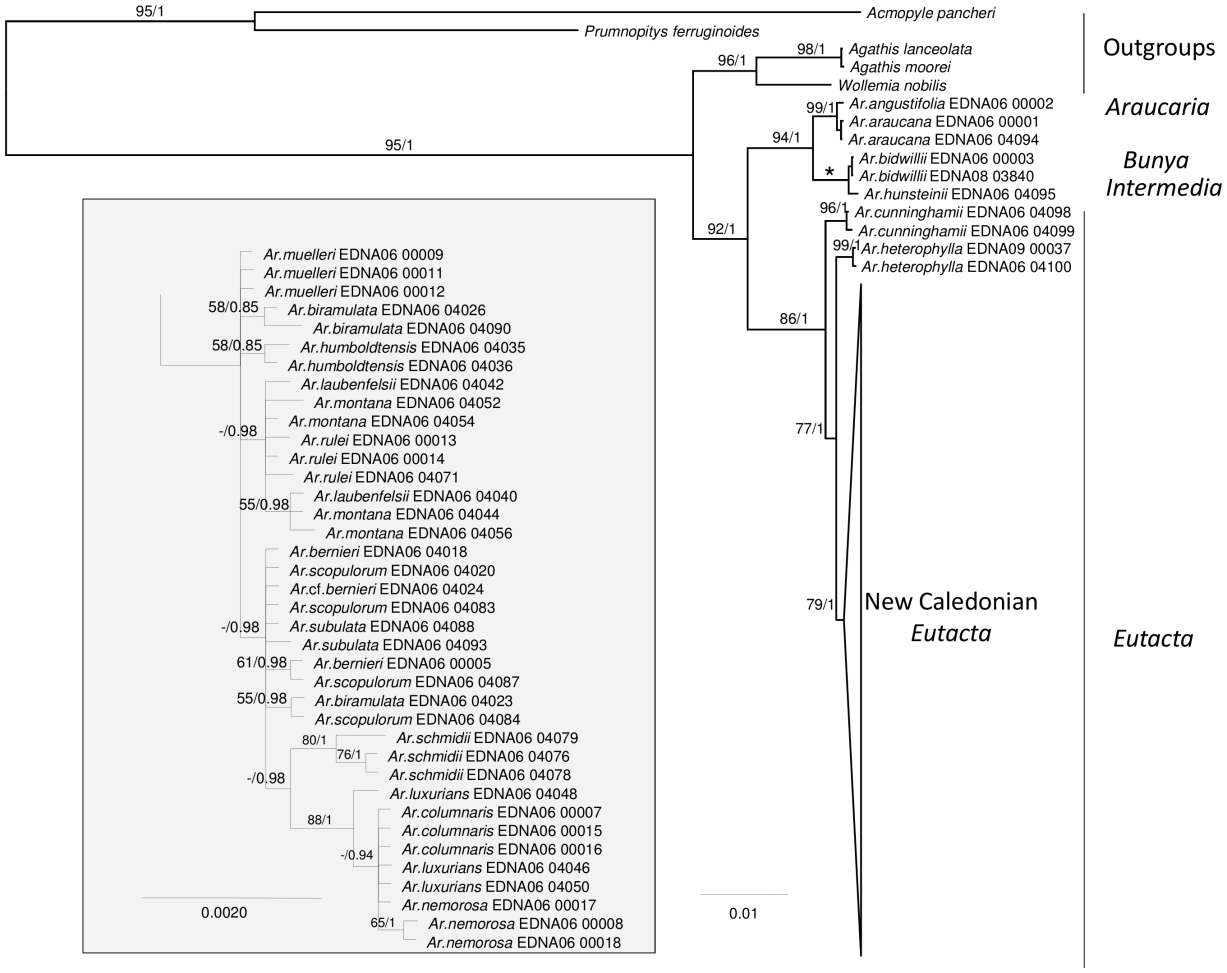
Araucariaceae phylogeny inferred from plastid DNA sequences using Bayesian optimisation criteria (50% majority rule topology). Support values, below the branch, are: ML bootstrap/Bayesian posterior probability. Branches marked with an asterisk have a BS of 100% and a PP of 1.0. Detail of the relationships among New Caledonian *Araucaria* is shown at the bottom left. Branch lengths are proportional to the inferred number of substitutions along that branch.

**Figure 3:**
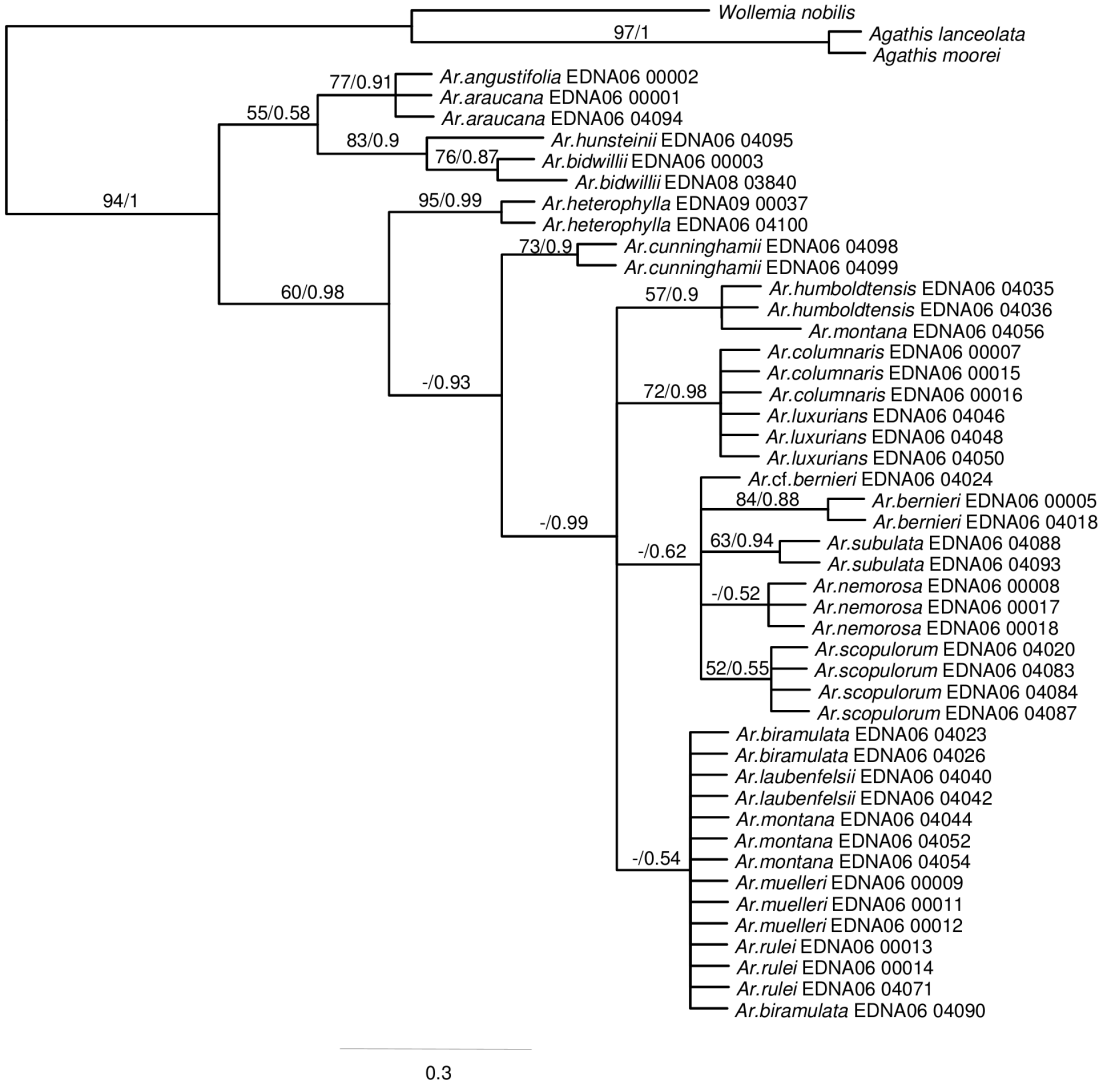
Araucariaceae phylogeny inferred from ITS2 DNA sequences using Bayesian optimisation criteria (50% majority rule topology). Support values are as detailed for Figure 2.

**Figure 4:**
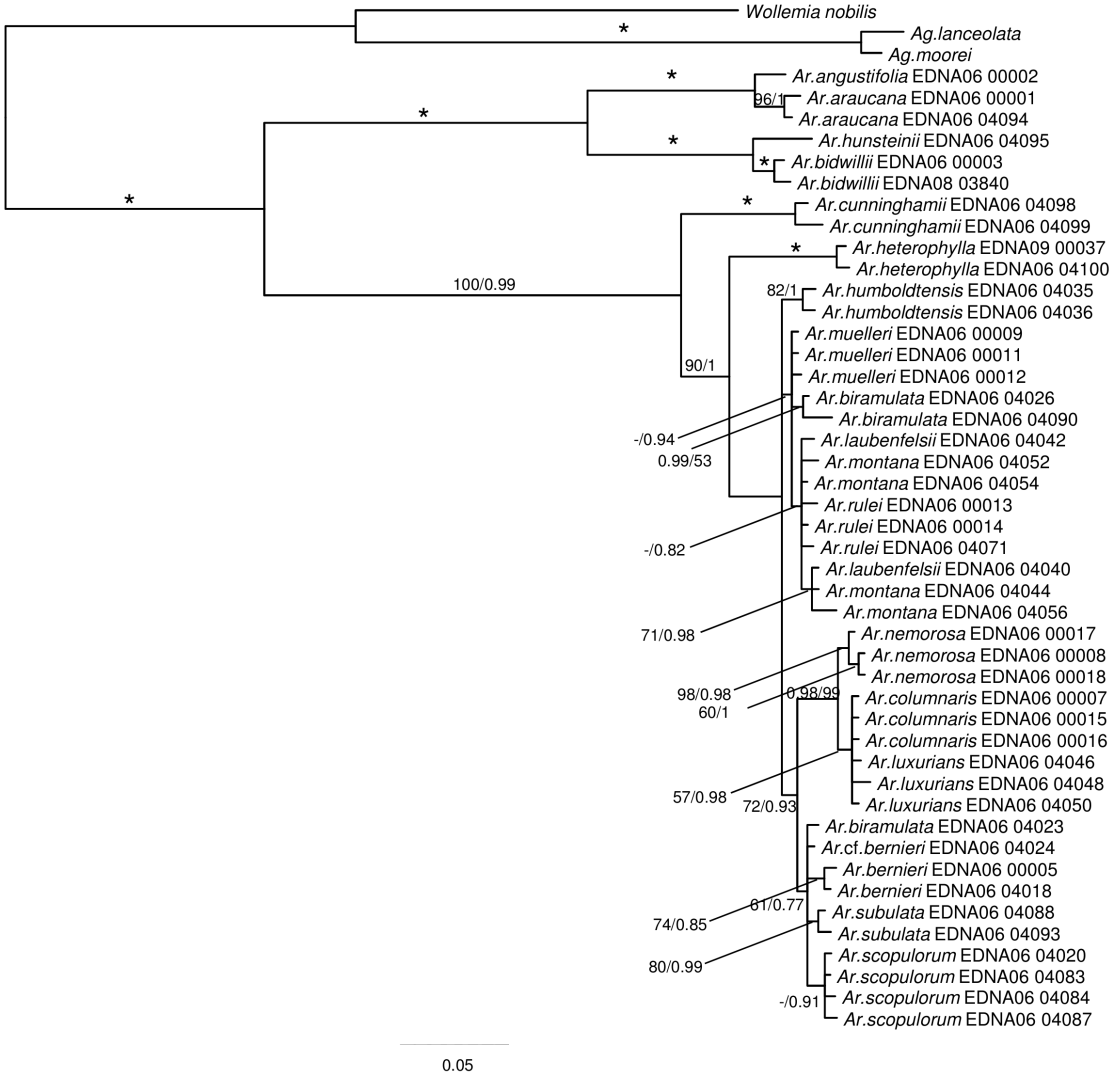
Araucariaceae phylogeny inferred from a combined plastid and ITS2 DNA data set using Bayesian optimisation criteria (50% majority rule). Support values are as detailed for Figure 2.

Phylogenies estimated from the plastid (Figure 2) and ITS2 (Figure 3) data resolved broadly similar groupings including *Wollemia* + *Agathis* and *Araucaria*. Within *Araucaria*, section *Eutacta* is resolved as sister to a clade including the sections *Araucaria* and *Bunya* Wilde and Eames + *Intermedia* C.T.White. ITS2 data (Figure 3) resolved *A. heterophylla* (Salisb.) Franco as sister to the remaining *A*. section *Eutacta* clade, but this placement is relatively weakly supported (BS<50%; PP = 0.93). In the combined plastid and ITS2 data, there is robust support for *A. cunninghamii* Mudie as sister to the Pacific *A*. section *Eutacta* (*A. heterophylla*+New Caledonian species) (Figure 4). There is strong support for the monophyly of New Caledonian *A*. section *Eutacta*, but low resolution within this clade. In the combined data, well-supported groupings (here, defined as having a BS≥75% and PP≥0.95) include *A. nemorosa* de Laub. + *A. luxurians* (Brongn. and Gris.) de Laub. + *A. columnaris* (G.Forst.) Hook., and multiple accessions of some species including *A. nemorosa, A. humboldtensis* J.Buchholz and *A. subulata* Vieill. In the plastid data set, the three accessions of *A. schmidii* are also resolved with strong support, but were not included in the combined analyses because ITS2 sequences could not be obtained (Figures 2–4).

### Molecular dating of Araucariaceae

#### Assessment of fossil constraints

We used an expanded sampling of taxa and four plastid loci to infer araucarian phylogeny using a BRC model (Figure 1). With respect to relationships, these data are largely consistent with the results reported above.

**Figure 5:**
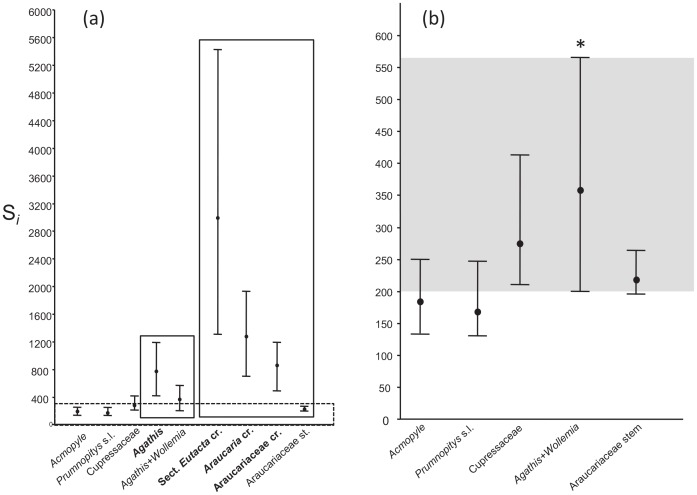
Assessment of calibration constraints using empirical scaling factor (S*_i_*) estimates. The mean (circle) and 95% highest posterior density interval (lines) of S*_i_* is shown for each proposed calibration, including: (a) alternative nodal placements of fossil *Agathis* and *Araucaria* (vertical boxes). In (a), the S*_i_* intervals are contrasted with the estimated age of conifers (c. 320 Ma; horizontal box). In (b), candidate fossils with S*_i_* estimates that consistently exceeded the maximum age of conifers have been removed. The grey shading indicates the region of overlap in S*_i_* intervals for the five retained calibrations.

Scaling factor distributions estimated under a BRC approach with unconstrained branch lengths are shown in Figure 5. Of the potential calibrations tested, the highest S*_i_* estimates were associated with the ‘*Eutacta*’ fossil constraint, although assigning an Early Jurassic age to the *Araucaria* section *Eutacta* crown, *Araucaria* crown or Araucariaceae crown (nodes 6, 7 and 8, respectively; Table 1) results in a distribution of S*_i_* values that consistently exceeds the age of the earliest known conifer fossils (i.e.> c. 310 Ma), or indeed, the oldest known land plants. Similarly, constraining the *Agathis* crown node to a Late Paleocene age returned unrealistic S*_i_* estimates. After excluding these outliers, we identified the constraint on node 5 as returning the highest empirical scaling factor, and 4 potential clade age calibrations (fossils associated with nodes 1, 2 3 and 9) with S*_i_* intervals that substantially overlap with node 5.

#### Araucarian divergence times

Five fossil constraints with overlapping *S_i_* intervals (Figure 5; Table 1) were used to estimate araucarian divergence times, varying the mean of the log normal calibrations priors to model taphonomic bias. In general, node age estimates substantially overlapped, irrespective of the log normal prior mean but differed substantially in terms of the upper versus lower values encompassed within the estimated 95% HPD. As there is no strong basis to favour a particular distribution of node age priors, we consider the full range of posterior value estimates as equally plausible (Table 2). Our estimates for the Araucariaceae stem (node 9) range from c. 284–202 Ma; the Araucariaceae crown (node 8), c. 138–60 Ma; the MRCA of *Agathis* + *Wollemia* (node 5) is estimated to have diversified at c. 91–55 Ma, while the *Agathis* crown (node 4) ranges from c. 55–12 Ma. The *Araucaria* crown (node 7) is estimated to have diverged c.103–29 Ma and our analyses support a preponderance of Cenozoic radiations among extant *Araucaria*. For instance, the MRCA of sections *Araucaria, Bunya* and *Intermedia* radiated between c. 58–9 Ma, while the diversification of crown *Eutacta* (node 6) is estimated at between c. 51–9 Ma. The radiation of the Pacific *Eutacta* (MRCA *A. heterophylla* + New Caledonian *Eutacta*) is Oligocene-Miocene age (c. 33–5 Ma), and the New Caledonian species appear to have diversified from the Miocene-Pliocene (c. 19–3 Ma) (Table 2).

**Table 2:** Node age estimates (millions of years) for Araucariaceae estimated from the BRC analyses of DNA sequences (Prior 1, log normal prior mean  =  minimum fossil age +10%; Prior 2, log normal prior mean  =  minimum fossil age +50%).

node	age estimate prior 1 (median[95%HPD])	age estimate prior 2 (median[95%HPD])	range
node 4 (*Agathis* crown)	26 (47–12)	31 (55–13)	**55–12**
node 5 (*Agathis*+*Wollemia*)	61 (77–55)	72 (91–60)	**91–55**
node 6 (*Eutacta* crown)	21 (43–9)	25 (51–11)	**51–9**
node 7 (*Araucaria* crown)	55 (87–29)	64 (103–34)	**103–29**
node 8 (Araucariaceae crown)	81 (122–60)	94 (138–68)	**138–60**
node 9 (Araucariaceae stem)	225 (233–202)	250 (284–224)	**284–202**
Pacific *Eutacta* (*A. heterophylla*+New Caledonia)	14 (28–5)	16 (33–7)	**33–5**
New Caledonian *Eutacta*	7 (15–3)	9 (19–3)	**19–3**

The preferred node age estimates (values in bold) encompass the full range of values included in the 95% HPD of both prior scenarios. All tabulated nodes are supported by a PP of 1.0.

## Discussion

### Phylogenetic relationships

Several studies have used molecular data to infer Araucariaceae phylogeny [8], [11], [15], [17]–[19], [47]. These are generally consistent regarding relationships among genera and among the major lineages within *Araucaria*. While the New Caledonian species of *Araucaria* section *Eutacta* consistently form a clade, establishing a robust understanding of interspecific relationships has proven difficult [7], [8], [37]. AFLP markers resolved three main genetic groups including a small leaved, large leaved and a coastal species group [7]. Escapa and Catalano [11] presented an analysis of a combined morphological and molecular data set, and found reasonable agreement with these interspecific groupings but their hypothesis differed from Gaudeul et al. [7] in the resolution of group inter-relationships. In the present study, employing a relatively large amount of sequence data (Table S1) did return some well supported resolutions among the New Caledonian *Araucaria* including the coastal group (*A. nemorosa, A. luxurians, A. columnaris*) as noted above. However, additional lines of evidence are clearly required to more fully resolve relationships and to test the monophyletic status of some of the species (Figures 3 and 4). Low levels of genetic divergence in previous studies and ours argue for a relatively recent and rapid radiation of the New Caledonian species [2], [7], [8].

### Assessment of fossil constraints

Molecular dating has become a routine approach in molecular phylogenetic studies, and is central to evolutionary interpretation [16]. While there have been several advances in molecular clock methods, a key issue is the placement of fossil constraints, which, regardless of the clock methods employed, can strongly influence branch lengths. Several recent studies have used cross-validation approaches to identify sets of internally consistent constraints [38], [48], [49].

The fossil record of Araucariaceae extends to the Triassic, although in many cases the affinities of pre-Jurassic remains are doubtful [32]. Nevertheless, several fossil types from the Early Mesozoic have been assigned to extant araucarian lineages [9], [10], [33]. In the light of our analyses using unconstrained branch lengths, we find little support for this approach: assigning an Early Jurassic age to the *Araucaria* section *Eutacta* crown, *Araucaria* crown or Araucariaceae crown (nodes 6, 7 and 8, respectively; Table 1) results in a distribution of S*_i_* values that are unrealistic in the context of the evolutionary time scale of land plants [50]. We interpret these values as reflecting incorrect phylogenetic placement of the ‘*Eutacta*’ fossil constraint which would bias age estimates to be too old if retained in molecular clock analyses. Indeed, Biffin et al. [15] found that minimally constraining *Araucaria* to a Middle Cretaceous age resulted in implausibly high levels of molecular rate variation between stem and crown Araucariaceae or alternatively, unrealistically old estimates for the divergence of extant conifers. In the present study, assuming an Early Jurassic age for stem Araucariaceae (node 9) returned a distribution of S*_i_* estimates that substantially overlapped with the intervals for four other fossils, and in light of the conifer fossil record, provides a credible estimate of the age of the root [18] (Figures 1 and 5; Table 1).

### Palaeobotanical implications

If the *Araucaria* crown group radiated not more than c. 100 Ma (Figure 1; Table 2) this casts doubt on the status of several relatively ancient fossil types with putative affinities to extant taxa (e.g. Axsmith et al., 2008). However, aspects of this record have previously been questioned on a number of grounds. Firstly, several authors have noted that the extant diversity of Araucariaceae almost certainly underestimates past diversity and morphological variation within the family [8], [10], [42], [51]. In this context, araucarian fossils have been described that possess unique combinations of character states but these overlap with extant variation, e.g. *Yezonia* Stopes and Fujii [52], making the placement of fragmentary fossil material problematic. Secondly, in the light of the discovery of *Wollemia* and its phylogenetic placement in the family, it has been suggested that important characters used to diagnose *Araucaria* and its extant sections may be plesiomorphic for the family [11], [42], [51]. Thirdly, the low levels of molecular sequence divergence among extant *Araucaria* are difficult to reconcile with a Jurassic fossil record for these lineages [15]. Taken together, it has been suggested that the pre-Cretaceous fossil record of putative *Araucaria* may entirely represent stem Araucariaceae, or (at best) stem lineages of the extant genera [11], [42]. However, the molecular dates presented here are not inconsistent with a hypothesis that crown *Araucaria* had evolved by the late Early Cretaceous as indicated by fossil taxa assigned to living sections, e.g. *A. grandiflora* Feruglio and *A. otwayensis* Cantrill, estimated at c. 105–95 Ma from Patagonia and southern Australia and referred to sections *Araucaria* and *Eutacta*, respectively [33], [53], [54] (Table 2, Figure 1).

The fossil record for the crown *Agathis* clade (i.e. *Agathis+Wollemia*) extends from the Late Cretaceous in reasonable agreement with our molecular age estimates for the corresponding nodes (Figure 1, Table 2). While there are no formal descriptions from the macrofossil record, pollen that is almost indistinguishable from that of extant *Wollemia nobilis* (*Dilwynites* W.K.Harris sp.) is first recorded in Turonian (c. 93–89 Ma) strata of Australia and the Maastrichtian (c. 70–65 Ma) of New Zealand, representing the oldest probable record of that genus [51] (but see [55]). In a recent review of the macrofossil record of *Agathis* it was concluded that the oldest unequivocal remains for that genus are from the Late Palaeocene (c. 55–50 Ma) of Southern Australia [30]. Putative *Agathis* macrofossils have been recorded from the Cretaceous but lack organic preservation, e.g. *A. victoriensis* Cantrill of the Lower Cretaceous, c. 110 Ma, of Southern Australia [53] or include characters that are atypical of modern *Agathis* [56], [57] and therefore remain equivocal [30]. Using a cladistic analysis of morphological characters, the fossil taxa *Wairarapaia mildenhallii* Cantrill and Raine, and *Emwadea macrocarpa* Dettmann, Clifford and Peters, were resolved as stem group lineages of the *Agathis-Wollemia* clade [11] and date from the Early Cretaceous of New Zealand and Australia, respectively [42], [58].

### Biogeographic implications

The present distribution of *Araucaria*, which is Gondwanan in character, is often interpreted as relictual, reflecting regional (Northern Hemisphere) extinction, localised (Southern Hemisphere) contraction and Gondwanan tectonic events [9], [10]. Our molecular dating scenario is consistent with aspects of this view. For instance, there is evidence for the presence of *Araucaria* in Europe until c. 66 Ma, which has been proposed as the youngest reliable record of the genus for the Northern Hemisphere [59]. In light of our findings, this date does not reject placement of these fossils within crown *Araucaria* (Figure 1) although in contrast to some interpretations of the Northern Hemisphere fossil record, our dated phylogeny suggests that the crown groups of the extant sections are derived entirely from Cenozoic Southern Hemisphere radiations. The regional (Northern Hemisphere) extinction of Araucariaceae was probably multicausal [10]. It has been linked to palaeoclimatic and vegetation change [59] as well as to short term environmental perturbations at the Cretaceous-Palaeogene (K-Pg) boundary (c. 65 Ma) following the asteroid impact at Yucatan (see also [34] where the virtual disappearance of *Araucaria* at the K-Pg boundary of New Zealand is noted). A recent study documents the survival of a seed fern lineage (*Komlopteris* Barbacka) into the Eocene of Tasmania [60]. These authors hypothesise that a delayed radiation of the angiosperms at high latitudes, and the remoteness of southern Gondwana from the Yucatan impact site may have afforded refuge to *Komlopteris* and other ‘archaic’ gymnosperm lineages into the Cenozoic [60]. Given this hypothesis, *Araucaria*, too might have persisted in isolated Gondwanan refuges following the K-Pg boundary event and radiated from these during the Palaeocene [34]. In this case, one implication is that the pre-Cretaceous Northern Hemisphere fossil record of Araucariaceae may have little direct bearing on the evolution of extant *Araucaria*.

The importance of Gondwanan tectonic vicariance versus long distance dispersal in the assembly of the Southern Hemisphere flora has been widely debated and generally, the latter appears to be prominent [61]. Dispersal is implicated by clade origination times (i.e. stem group age estimates) that are younger than the timing of geological events [5]. In the context of our data, vicariance is not rejected for the divergence of the clade that includes *Araucaria* section *Araucaria* (southern South America) and sections *Bunya* + *Intermedia* (Australia and New Guinea, respectively). Southern South America and Australia remained connected via Antarctica until the Neogene [62], [63] and potentially harboured a widespread ancestor of these lineages [64].

The biogeography of New Caledonia has been considered as an example where geological and biological evidence are at odds [2], [5], [6], [65]. New Caledonia supports an apparently ancient biota [3] despite the lack of firm geological evidence for a continuously emergent land surface following the Late Cretaceous-Palaeogene rifting of New Caledonia (as part of the continental fragment, Zealandia) from East Gondwana [4], [65]. The re-emergence of New Caledonia c. 37 Ma places an upper limit on the age of the island's biota. However, the radiation of *Araucaria* section *Eutacta* on New Caledonia may be younger still if the emersion of Norfolk Island (c. 3.7 Ma) is assumed to constrain the possible age of Pacific *Araucaria* section *Eutacta* [2] (Figures 1 and 4). In the present study, we find that the relevant divergence (c. 33–5 Ma) is too old to support this hypothesis and implicitly rejects a single long distance dispersal event from Australia to Norfolk Island for the MRCA of Pacific *Araucaria* section *Eutacta*. Alternative plausible scenarios for the origin of *A. heterophylla* involve a dispersal event from Australia to New Caledonia and a subsequent dispersal from either New Caledonia or Australia. In either case, the second dispersal event requires an extinction event post-dating the emersion of Norfolk Island involving the *A. heterophylla* lineage in New Caledonia, or the Pacific *Araucaria* section *Eutacta* lineage in Australia. Given the uncertainty in divergence time estimates for the *Eutacta* crown (51-9 Ma), the origin of New Caledonian *Araucaria* could be explained by a single dispersal or alternatively by short range dispersals among ephemeral landmasses such as those identified or are believed to have existed on the South Norfolk Rise [66], [67] and in the Greater New Caledonia region [4], [14]. In either case, our data do reject a ‘museum’ type hypothesis for New Caledonian *Araucaria* and implicate at least some over water dispersal, given that the New Caledonian endemic clade has arisen entirely after the estimated timing of the Palaeocene-Eocene marine transgressions and has radiated in the Neogene (c. 19-3 Ma; Table 2, Figure 1).

## Supporting Information

Table S1 **Accession details of individuals used in the phylogenetic analyses.** Country abbreviations in the ‘Location’ column are AU…Australia, AR…Argentina, CL…Chile, NC…New Caledonia. Superscripts denote cultivated material: 1 Royal Botanic Garden Edinburgh (UK), 2 Mount Lofty Botanic Garden (AU), 3 Koishikawa Botanic Garden (Japan), 4 Adelaide Botanic Garden (AU). Voucher numbers refer to herbarium specimens deposited in Edinburgh (E), University of Adelaide (ADU) or Allan Herbarium, Christchurch (CHR).
(DOCX)

Table S2 **Genbank accession numbers of all samples used for the phylogenetic analyses.**
(XLSX)
