# Supplementary material for: Evolutionary Diversification of New Caledonian Araucaria
Source: PLoS One. 2014 Oct 23;9(10):e110308. doi: 10.1371/journal.pone.0110308 (PMC4207703; doi:10.1371/journal.pone.0110308)
Supplement: Table S1 — Accession details of individuals used in the phylogenetic analyses. Country abbreviations in the ‘Location’ column are AU…Australia, AR…Argentina, CL…Chile, NC…New Caledonia. Superscripts denote cultivated material: 1 Royal Botanic Garden Edinburgh (UK), 2 Mount Lofty Botanic Garden (AU), 3 Koishikawa Botanic Garden (Japan), 4 Adelaide Botanic Garden (AU). Voucher numbers refer to herbarium specimens deposited in Edinburgh (E), University of Adelaide (ADU) or Allan Herbarium, Christchurch (CHR). (DOCX) [file pone.0110308.s001.docx]

Table S1. Accession details of individuals used in the phylogenetic analyses. Country abbreviations in the ‘Location’ column are AU…Australia, AR…Argentina, CL…Chile, NC…New Caledonia. Superscripts denote cultivated material: ^1^ Royal Botanic Garden Edinburgh (UK), ^2^ Mount Lofty Botanic Garden (AU), ^3^ Koishikawa Botanic Garden (Japan), ^4^ Adelaide Botanic Garden (AU). Voucher numbers refer to herbarium specimens deposited in Edinburgh (E), University of Adelaide (ADU) or Allan Herbarium, Christchurch (CHR).

| Species | EDNA number | Collection or accession number | Location | Voucher | Permit issuer |
| --- | --- | --- | --- | --- | --- |
| *Acmopyle pancheri* | EDNA08_02784 | NC05_73 | 22.119S/166.658E  Parc Territorial de la Rivière Bleue, NC | E00215108 | Province Sud, NC |
| *Prumnopitys ferruginoides* | EDNA06_01761 | NC05_100 | 21.881S/166.419E  Mont Humboldt, NC | E00215020 | Province Sud, NC |
| *Agathis lanceolata* | EDNA06_04097 | CAGNC_24 | 22.212S/166.667E  Col de Mouirange, NC | E00107144  E00107145 | Province Sud, NC |
| *Agathis atropurpurea*^2^ | n/a | n/a | not of known wild origin | n/a | Botanic Garden |
| *Agathis australis*^2^ | n/a | G874336 | not of known wild origin | n/a | Botanic Garden |
| *Agathis borneensis*^1^ | n/a | 19622877 | not of known wild origin | n/a | Botanic Garden |
| *Agathis endertii*^1^ | n/a | 19820755 | not of known wild origin | n/a | Botanic Garden |
| *Agathis macrophylla*^1^ | n/a | 19860867 | not of known wild origin | n/a | Botanic Garden |
| *Agathis microstachya*^1^ | n/a | 20030755 | not of known wild origin | n/a | Botanic Garden |
| *Agathis montana*^2^ | n/a | W990969 | not of known wild origin | n/a | Botanic Garden |
| *Agathis moorei* | EDNA06_04096 | CAGNC_71 | 20.407S/ 164.525E  Road to Mandjélia, NC | E00106192 | Province Nord, NC |
| *Agathis obtusa*^1^ | n/a | 19860865 | not of known wild origin | n/a | Botanic Garden |
| *Agathis robusta*^2^ | n/a | G834223 | not of known wild origin | n/a | Botanic Garden |
| *Wollemia nobilis*^1^ | EDNA08_03022 | 20042112 | not of known wild origin | E00328837 | Botanic Garden |
| *Araucaria angustifolia*^3^ | EDNA06_00002 | AM001539 | not of known wild origin | E00131696 | Botanic Garden |
| *Araucaria araucana*^1^ | EDNA06_00001 | 19990981 | 37.862S/71.072W  Caviahue - Fragment 4, AR | n/a | Botanic Garden |
|  | EDNA06_04094 | 19990741 | 37.08S/73.00W  Nahuelbuta National Park, CL | n/a | Botanic Garden |
| *Araucaria bidwillii*^1^ | EDNA06_00003 | 19841690 | not of known wild origin | n/a | Botanic Garden |
| *Araucaria bidwillii* | EDNA08_03840 | n/a | 16'.60S/145.27’E  Mount Lewis National Park, AU | E00210626 | National Park Authority |
| *Araucaria cunninghamii*^1^ | EDNA06_04098 | 19762124 | 13.70S/143.32E  NE of Coen, AU | E00127974 | Botanic Garden |
|  | EDNA06_04099 | 19762122 | 21.75S/149.08E  Tierawoomba, AU | E00127973 | Botanic Garden |
| *Araucaria heterophylla*^4^ | EDNA09_00037 | n/a | not of known wild origin | n/a | Botanic Garden |
| *Araucaria heterophylla*^1^ | EDNA06_04100 | 19885028 | not of known wild origin | n/a | Botanic Garden |
| *Araucaria hunsteinii*^1^ | EDNA06_04095 | 19623223 | 7.33S/146.70E  near Wau, Papua New Guinea | E00127992 | Botanic Garden |
| *Araucaria bernieri* | EDNA06_00005 | NC01_669 | 22.25S/166.82E  Pic des Pins, NC | E00137601 | Province Sud, NC |
|  | EDNA06_04018 | NC03_4251 | 22.12S/160.60E  Montagne des Sources, NC | E00166481 | Province Sud, NC |
| *Araucaria* cf. *bernieri* | EDNA06_04024 | NC03_4261 | 22.12S/160.60E  Montagne des Sources, NC | E00131781 | Province Sud, NC |
| *Araucaria biramulata* | EDNA06_04023 | NC03_4090 | 21.76S/166.00E  Mt Do, NC | E00166497 | Province Sud, NC |
|  | EDNA06_04026 | NC05_185 | 21.93S/166.25E  Mont Tonta, NC | E00215078 | Province Sud, NC |
|  | EDNA06_04090 | NC05_6 | 22.27S/166.90E  Pic du Grand Kaori, NC | E00215029 | Province Sud, NC |
| *Araucaria columnaris* | EDNA06_00007 | NC01_833 | 22.317S/167.012E  north of Rivière Tou, NC | E00137599 | Province Sud, NC |
|  | EDNA06_00015 | NC01_730 | 22.18S/166.85E  Port Boisé, NC | n/a | Province Sud, NC |
|  | EDNA06_00016 | NC01_5 | 21.60S/165.45E  Baie des Tortues, NC | E00137884 | Province Sud, NC |
| *Araucaria humboldtensis* | EDNA06_04035 | NC03_2007 | 22.12S/160.60E  Montagne des Sources, NC | E00131838 | Province Sud, NC |
|  | EDNA06_04036 | NC05_117 | 22.11S/166.89E  Mont Humboldt, NC | E00215065 | Province Sud, NC |
| *Araucaria laubenfelsii* | EDNA06_04040 | NC03_4055 | 21.76S/166.00E  Mt Do, NC | E00166495 | Province Sud, NC |
|  | EDNA06_04042 | NC01_601 | 22.07S/166.35E  Mt Mou, NC | E00137218 | Province Sud, NC |
| *Araucaria luxurians* | EDNA06_04046 | NC01_931 | 22.31S/166.68E  Plum, NC | E00137432 | Province Sud, NC |
|  | EDNA06_04048 | NC02_55 | 21.58S/165.83E  La Foa, NC | n/a | Province Sud, NC |
|  | EDNA06_04050 | NC03_4016 | 21.61S/166.23E  Botamere, NC | E00166500 | Province Sud, NC |
| *Araucaria montana* | EDNA06_04044 | NC03_4132 | 21.44S/165.85  Bwa Méyu, NC | E00166482 | Province Sud, NC |
|  | EDNA06_04052 | NC03_4243 | 20.56S/164.78E  Mt. Panié, NC | n/a | Province Nord, NC |
|  | EDNA06_04054 | NC02_46 | 20.85S/164.63E  Kopeto, NC | n/a | Province Nord, NC |
|  | EDNA06_04056 | NC05_260 | 21.28S/165.04E  Paéoua, NC | E00215092 | Province Nord, NC |
| *Araucaria muelleri* | EDNA06_00009 | NC01_653 | 22.25S/166.82E  Pic des Pins, NC | E00137598 | Province Sud, NC |
|  | EDNA06_00011 | NC03_3002 | 22.12S/160.60E  Montagne des Sources, NC | E00166515 | Province Sud, NC |
|  | EDNA06_00012 | NC01_870 | 22.27S/166.97E  Le Trou, NC | E00137600 | Province Sud, NC |
| *Araucaria nemorosa* | EDNA06_00008 | 366 | 22.35S/166.95E  Port Boisé, NC | E00210620 | Province Sud, NC |
|  | EDNA06_00017 | NC03_4013 | 22.30S/167.00E  Cap Reine Charlotte, NC | E00166501 | Province Sud, NC |
|  | EDNA06_00018 | 93 | 22.32S/166.93E  Forêt Nord, NC | n/a | Province Sud, NC |
| *Araucaria rulei* | EDNA06_00013 | NC01_312 | 21.77S/166.18E  Camp des Sapins, NC | E00141033 | Province Sud, NC |
|  | EDNA06_00014 | NC01_241 | 21.55S/166.05E  Bogota, NC | E00141268 | Province Nord, NC |
|  | EDNA06_04071 | NC01_39 | 20.48S/164.23E  Tiébaghi, NC | E00141840 | Province Nord, NC |
| *Araucaria schmidii* | EDNA06_04076 | NC03_4221 | 20.56S/164.78E  Mt. Panié, NC | n/a | Province Nord, NC |
|  | EDNA06_04078 | NC03_4228 | 20.56S/164.78E  Mt. Panié, NC | E00166491 | Province Nord, NC |
|  | EDNA06_04079 | NC03_4209 | 20.56S/164.78E  Mt. Panié, NC | n/a | Province Nord, NC |
| *Araucaria scopulorum* | EDNA06_04083 | NC02_851 | 20.28S/164.03E  Poum, NC | E00249004 | Province Nord, NC |
|  | EDNA06_04084 | NC01_271 | 21.55S/166.05E  Bogota, NC | E0014126 | Province Nord, NC |
|  | EDNA06_04087 | NC02_333 | 21.60S/166.20E  Thio, NC | E00210617 | Province Nord, NC |
|  | EDNA06_04020 | NC02_302 | 21.60S/166.20E  Thio, NC | E00210623 | Province Nord, NC |
| *Araucaria subulata* | EDNA06_04088 | NC01_679 | 22.03S/166.45E  Mt. Dzumac, NC | E00131569 | Province Sud, NC |
|  | EDNA06_04093 | NC05_69 | 22.120S/166.660E  Parc Territorial de la Rivière Bleue, NC | E00215105 | Province Sud, NC |
| *Dacrycarpus compactus*^1^ | n/a | 19643273 | 6.00S/146.00E  Sewe, Papa New Guinea | E00311916 | Botanic Garden |
| *Falcatifolium taxoides* | n/a | NC03_4039 | 22.10S/166.86E  Mamié, NC | E00119751 | Province Sud, NC |
| *Halocarpus kirkii*^2^ | n/a | G932291 | not of known wild origin | n/a | Botanic Garden |
| *Lepidothamnus fonkii*^1^ | n/a | 20060725 | not of known wild origin | n/a | Botanic Garden |
| *Phyllocladus alpinus* | n/a | Wardle 96.01 | 44.30S/169.42E  Lake Hawea, New Zealand | CHR Wardle 96.01 | Department of Conservation |
| *Podocarpus gnidioides*^1^ | n/a | 20010565 | 22.166S/166.515E  Mt Koghi, NC | E00311957 | Botanic Garden |
| *Retrophyllum rospigliosii*^1^ | n/a | 19951955 | not of known wild origin | E00311951 | Botanic Garden |
| *Sundacarpus amarus*^4^ | n/a | J.G.Conran 797A | not of known wild origin | ADU J.G.Conran 797A | Botanic Garden |
